# Localization of Ectopic Mediastinal Parathyroid Adenomas Using Indigo Carmine Injection for Surgical Management: A Preliminary Report

**DOI:** 10.3389/fsurg.2022.864255

**Published:** 2022-05-12

**Authors:** Makoto Kammori, Shinsaku Kanazawa, Hisae Ogata, Natsuki Kanda, Takashi Nagashima, Mahiro Kammori, Toshihisa Ogawa

**Affiliations:** ^1^Department of Breast and Endocrine Surgery, Niizashiki Central General Hospital, Saitama, Japan; ^2^Department of Breast Surgery, Koga Hospital, Shizuoka, Japan; ^3^Department of Laboratory, Niizashiki Central General Hospital, Saitama, Japan; ^4^School of Medicine, Shinshu University, Nagano, Japan; ^5^Department of Breast and Thyroid Surgery, Dokkyo University School of Medicine Saitama Medical Center, Saitama, Japan

**Keywords:** ectopic mediastinal parathyroid adenoma, enhanced CT scan, ^99m^sestamibi-MIBI scan, cervical ultrasound, indigo carmine blue injection, intraoperative parathyroid hormone assay

## Abstract

An ectopic parathyroid adenoma (EPA) is a rare entity. The aim of this study was to report our experience in the preoperative localization and surgical management of EPAs. This was a multicenter retrospective study involving patients diagnosed with an EPA (three males and seven females) from January 2005 to November 2021. The clinical features, preoperative management, and surgical procedures were analyzed. A cervical neck ultrasound was performed in all patients and showed a focus in eight patients. Cervicothoracic enhanced computed tomography was performed in all patients and showed a focus in nine patients. The ^99m^Tc-MIBI scintigraphy was performed in eight patients and showed uptake in six of them. We performed a neck dissection and thoracotomy in one patient, a thoracoscopy in one patient, surgery with a focused approach in seven patients, four of whom were injected with indigo carmine blue, and surgery with a bilateral approach in one patient. 1 h following the parathyroidectomy, the parathyroid hormone (PTH) concentration was decreased to 40–80% of the baseline value. Establishing a preoperative diagnosis of an EPA is challenging for the surgeon, despite the progress in the morphologic assessment. An intraoperative PTH assay and injection of indigo carmine have been shown to be valuable tools in the appropriate surgical management of an EPA.

## Introduction

The primary hyperparathyroidism (PHPT) is characterized by the autonomous secretion of parathyroid hormone (PTH) by one or more parathyroid glands. The most frequent cause of PHPT is a benign solitary parathyroid adenoma, which occurs in 80–90% of patients (1, 2). An ectopic focus is demonstrated in up to 22% of the patients; the prevalence is even higher in the patients who undergo redo surgery (3, 4). Surgery is the only definitive cure for PHPT and is indicated in all symptomatic patients, as well as a defined subgroup of asymptomatic patients (2, 5). The goals of parathyroid surgery are to remove all hyperfunctioning tissues and to preserve normal parathyroid tissues. In the past, the standard surgical parathyroid approach included bilateral cervical neck exploration. However, recently, a focused approach has gained wide acceptance as a safe and effective alternative. It is essential when adopting a focused approach to localize ectopic and supernumerary parathyroid glands. Thus, the pre- and intra-operative diagnoses must be guided by a surgical resection strategy to better identify the hyperfunctioning gland. The safety of the parathyroid staining with a methylene blue infusion has been established (6). Indigo carmine blue (5,5′-indigodisulfonic acid sodium salt) is an organic salt derived from indigo by aromatic sulfonation, which renders the compound soluble in water. During the renal function testing with indigo carmine blue, the excretion of indigo carmine from both ureters is observed with a cystoscope and the kidney status is assessed. Indigo carmine blue has also been used as a dye for sentinel lymph node biopsy during breast cancer surgery. The safety of indigo carmine blue injection has also been confirmed (7–10). The aim of our study was to evaluate our strategy in the pre-operative localization of ectopic parathyroid adenomas (EPAs), emphasizing the contribution of a dye method (i.e., indigo carmine blue injection) and an intraoperativeb PTH assay.

## Patients and Methods

**Figure 1:**
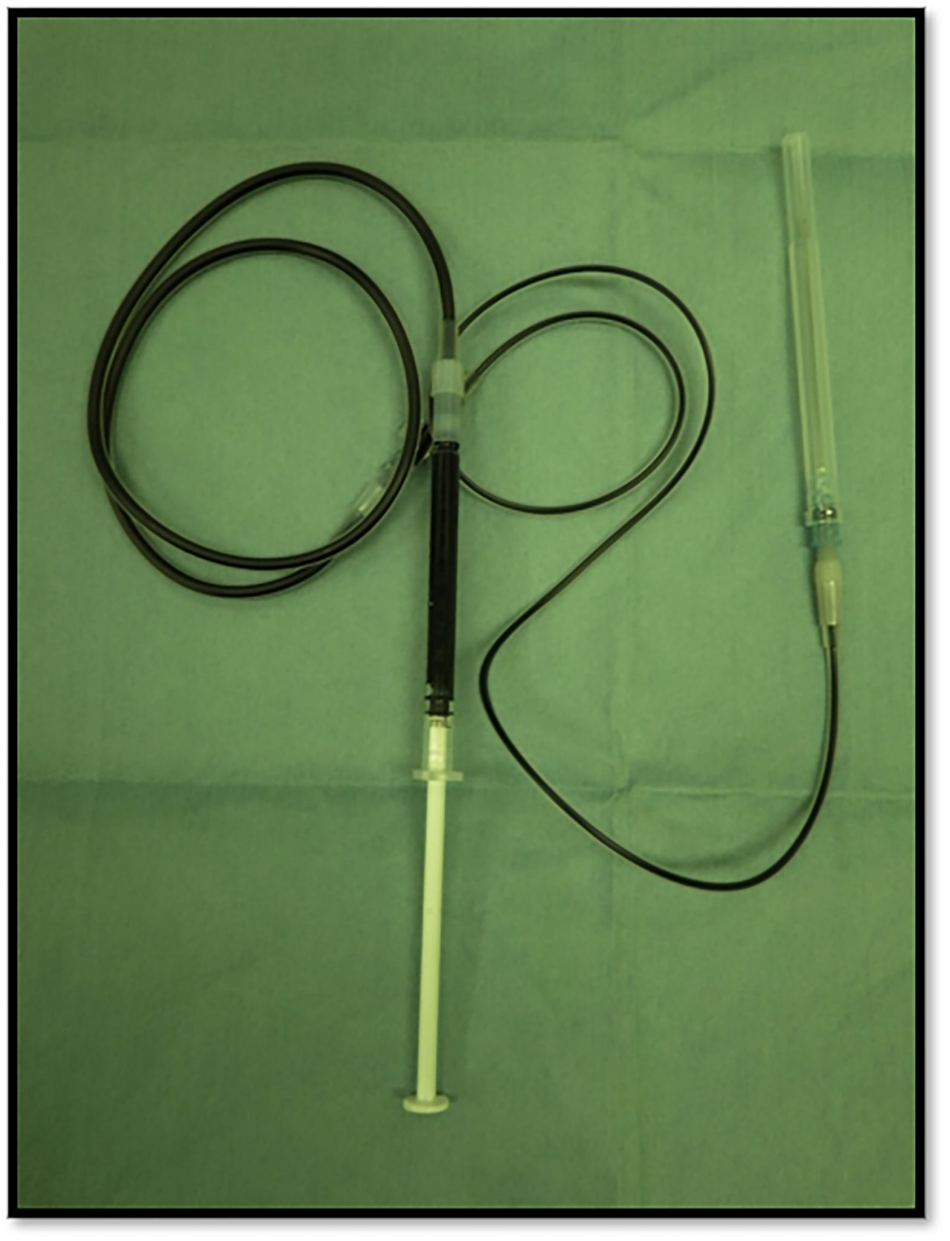
The indigo carmine injection set and indigo carmine dye-filled tube.

**Figure 2:**
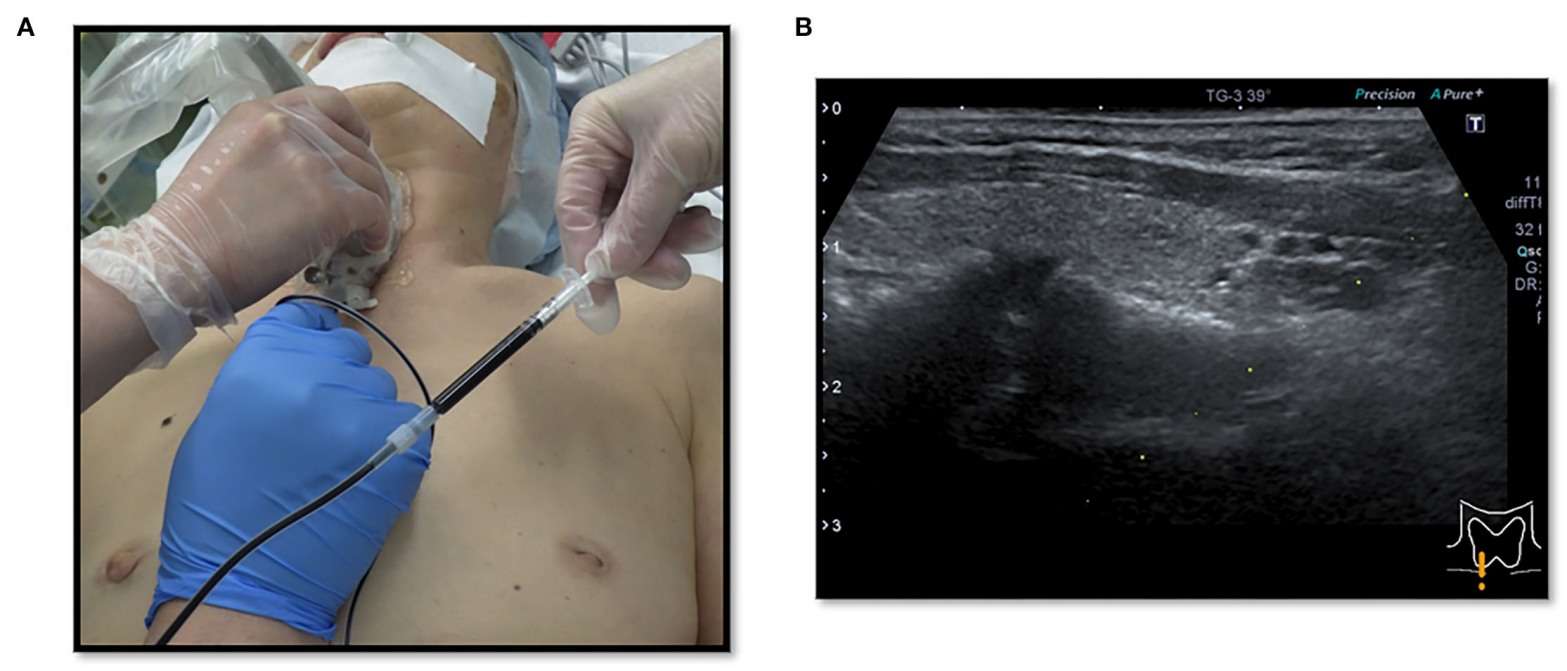
**(A,B)** Cervical neck ultrasound-guided injection of indigo carmine for marking EPA (dye method).

This study was based on the first author's (MK) cases. We conducted a multicenter (including the University Tokyo Hospital, Tokyo Women University Hospital, International University Health and Welfare Ichikawa Hospital, and Niizashiki Central General Hospital), retrospective study on patients managed for EPAs during the period extending from January 2005 to November 2021. We performed surgery on 139 patients (average age, 59.6 years) with PHPT and a parathyroid adenoma, and 14 patients (9.7%) with an ectopic lesion, among which 4 lesions (3.0%) were intra-thymic. Ten patients, excluding the 4 patients with intra-thymic lesions, were included in this study of EPAs. The normal parathyroid glands are supplied by the right and left inferior thyroid arteries on the dorsal aspect of the thyroid gland, and there are often four parathyroid glands above and below the arteries. Therefore, we decided to treat parathyroid adenomas as EPAs, in which blood flow was not clearly derived from the right and left inferior thyroid arteries by cervical ultrasonography. Our study was approved by the Institutional Ethics Committees of the International University Health and Welfare Ichikawa Hospital and the Niizashiki Central General Hospital. Since 2013, our management strategy for 7 patients was to perform the serum PTH assay intraoperatively. The assay is carried out in collaboration with the anesthesiologist team 1 h after resection of the parathyroid tumor. We performed preoperative intact-PTH measurements on the day before surgery. The surgical wound closure was performed after confirming a decrease in the intact PTH (i-PTH) concentration of at least 50% compared to the preoperative level. Since 2018, patients with EPAs were informed that the operation included an indigo carmine blue injection and informed consent was obtained. At our institution we use indigo carmine blue in sentinel lymph node biopsies for breast cancer, so we switched to methylene blue for EPA staining. Thus, we used indigo carmine as an alternative to methylene blue for the early identification and assessment of the parathyroid glands. We applied indigo carmine (Alfresa Pharma, Osaka, Japan) preoperatively to the lesion observed with cervical neck ultrasound. The EPA was visualized preoperatively by cervical neck ultrasound and cleaned with isopropyl alcohol. Indigo carmine [20 mg (5 ml)] was drawn into a 1-ml sterile injector (Figure 1). Approximately 0.3 ml of indigo carmine solution was prepared by dilution with a 23-gauge sterile needle and injected into the lesion with cervical neck ultrasound guidance (Figures 2A,B). Indigo carmine blue was injected under ultrasonography guidance 15 min before the operation began. We retrospectively reviewed the medical records of 10 patients admitted for surgical management of an EPA. The age, gender, details of detection, preoperative evaluation, choice of surgical approach, surgical procedure, and postoperative evaluation of patients were analyzed.

### Statistical Analysis

The significance of differences between tumor weight and a parameter of interest was analyzed using the Spearman rank correlation coefficient with statistical software EZR (Saitama Medical Center, Jichi Medical University, Saitama, Japan) (11). Differences at a *p* < 0.05 level were considered significant.

## Results

**Figure 3:**
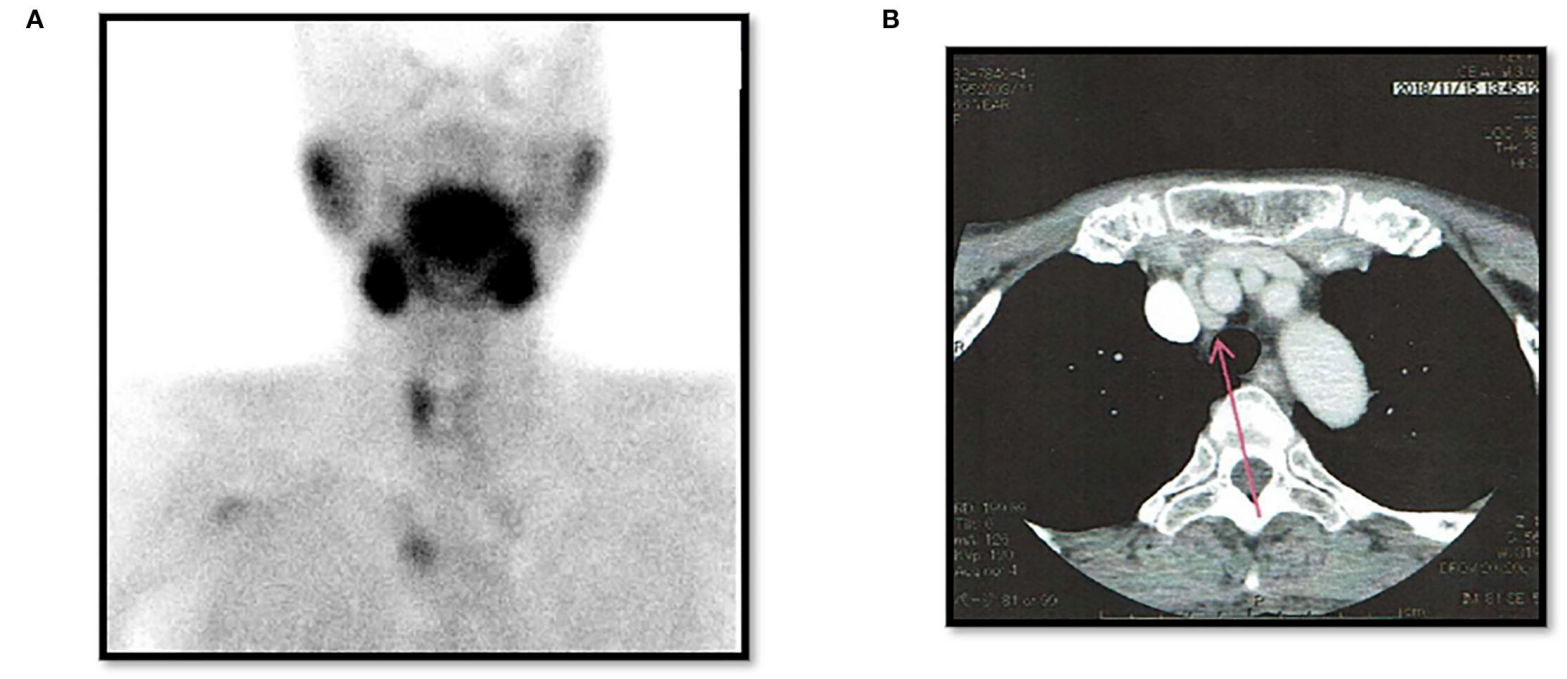
Case 3. Post-operative left thyroidectomy. **(A)** ^99m^Tc-MIBI (late phase) showing a right thyroid mass and anterior mediastinal washout staining (EPA). **(B)** Thoracic enhanced CT scan showing a mass between the right brachiocephalic artery and superior *vena cava* (EPA: red arrow).

**Figure 4:**
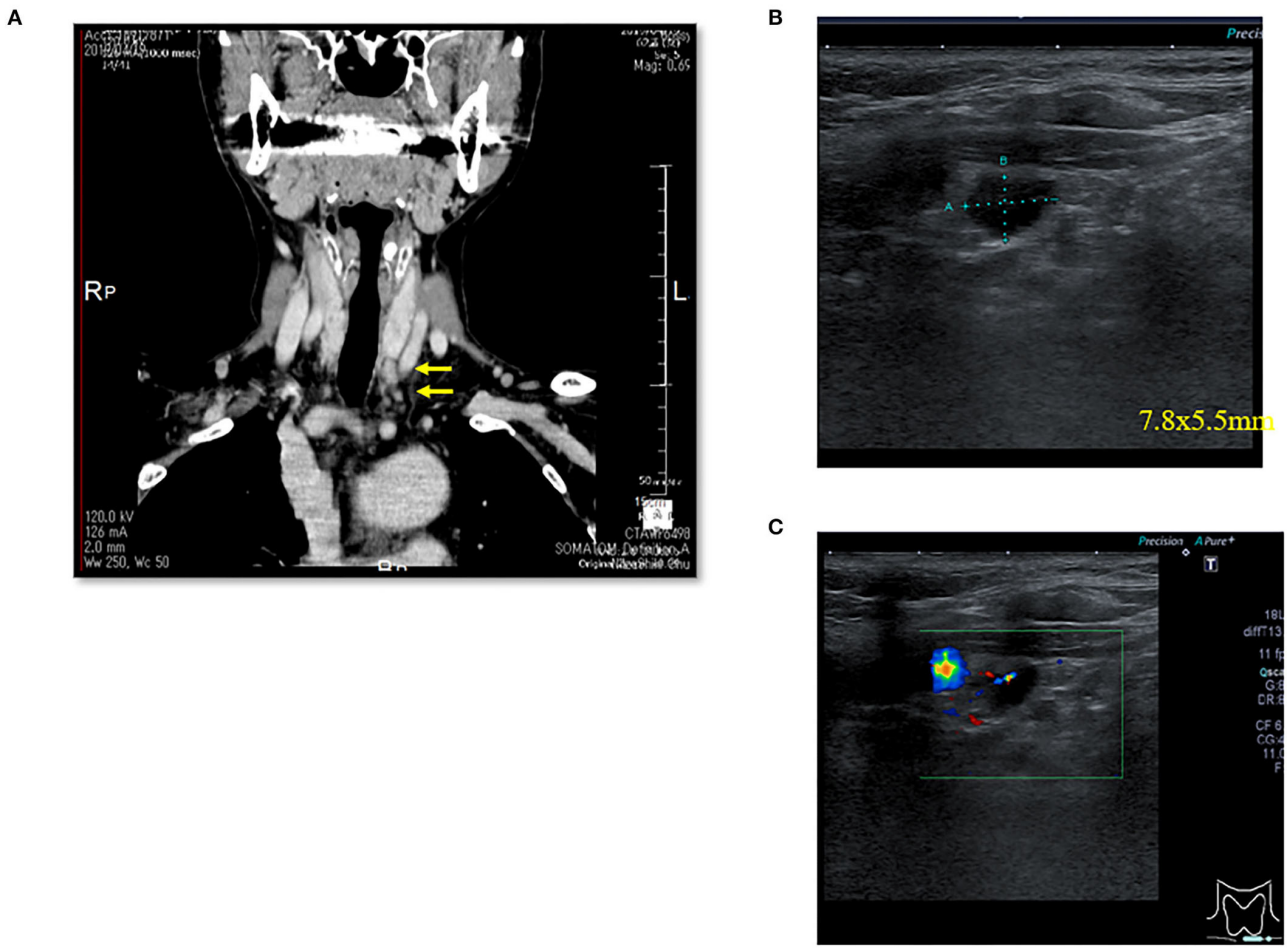
Case 4. **(A)** Thoracic enhanced CT (MPR) scan showing a left lower thyroid mass (EPA: yellow arrow). **(B)** Cervical neck ultrasound showing a left lower thyroid mass [7.8 × 5.5 mm (EPA)]. **(C)** Cervical neck ultrasound showing a left lower thyroid mass (EPA) and blood flow by color Doppler.

**Figure 5:**
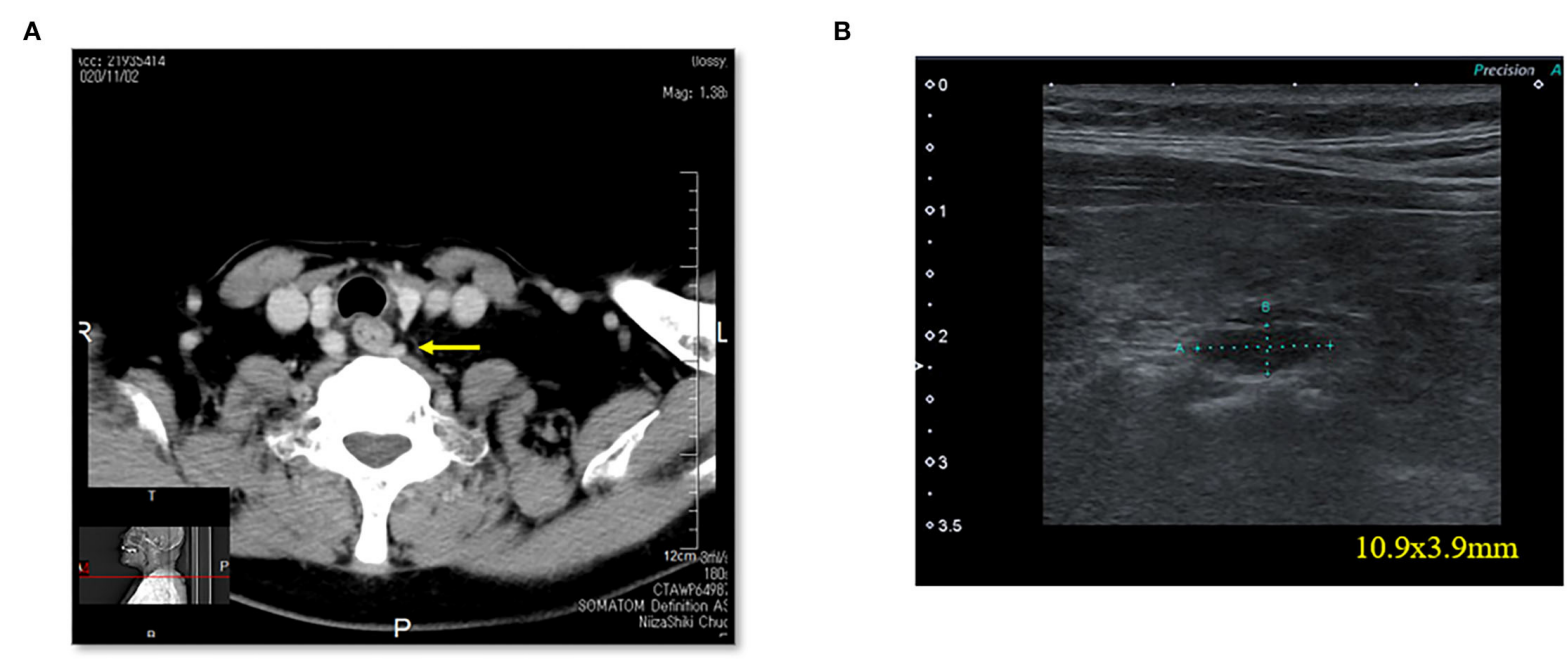
Case 6. **(A)** Thoracic enhanced CT scan showing a mass (EPA: yellow arrow) near the cervical esophagus. **(B)** Cervical neck ultrasound showing a mass (10.9 × 3.9 mm [EPA]) near the cervical esophagus.

**Figure 6:**
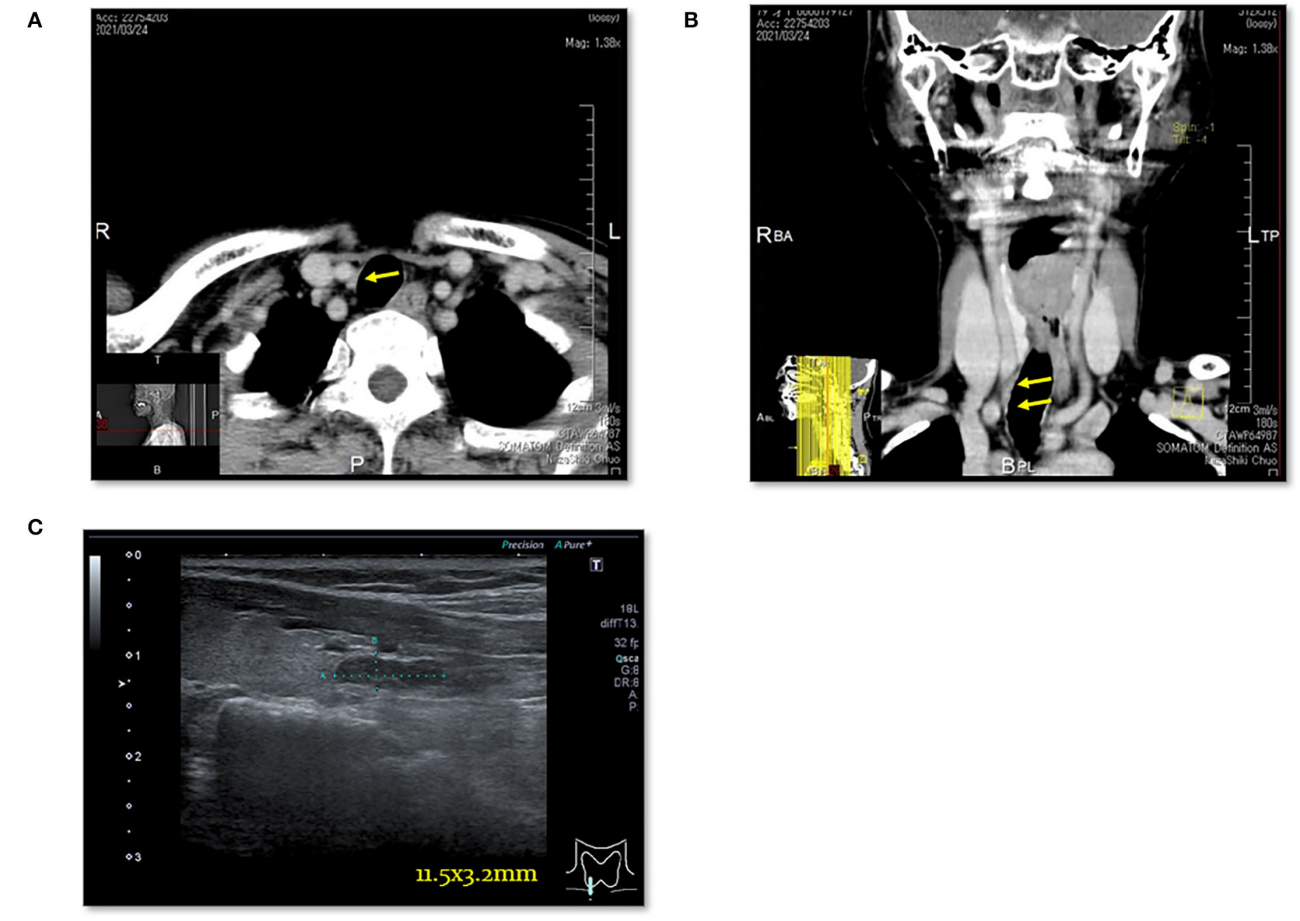
Case 8. **(A)** Thoracic enhanced CT scan showing a mass (EPA: yellow arrow) near the right trachea. **(B)** Thoracic enhanced CT (MPR) scan showing a mass (EPA: yellow arrow) near the right trachea. **(C)** Cervical neck ultrasound showing a mass [11.5 × 3.2 mm (EPA)] near the cervical esophagus.

**Figure 7:**
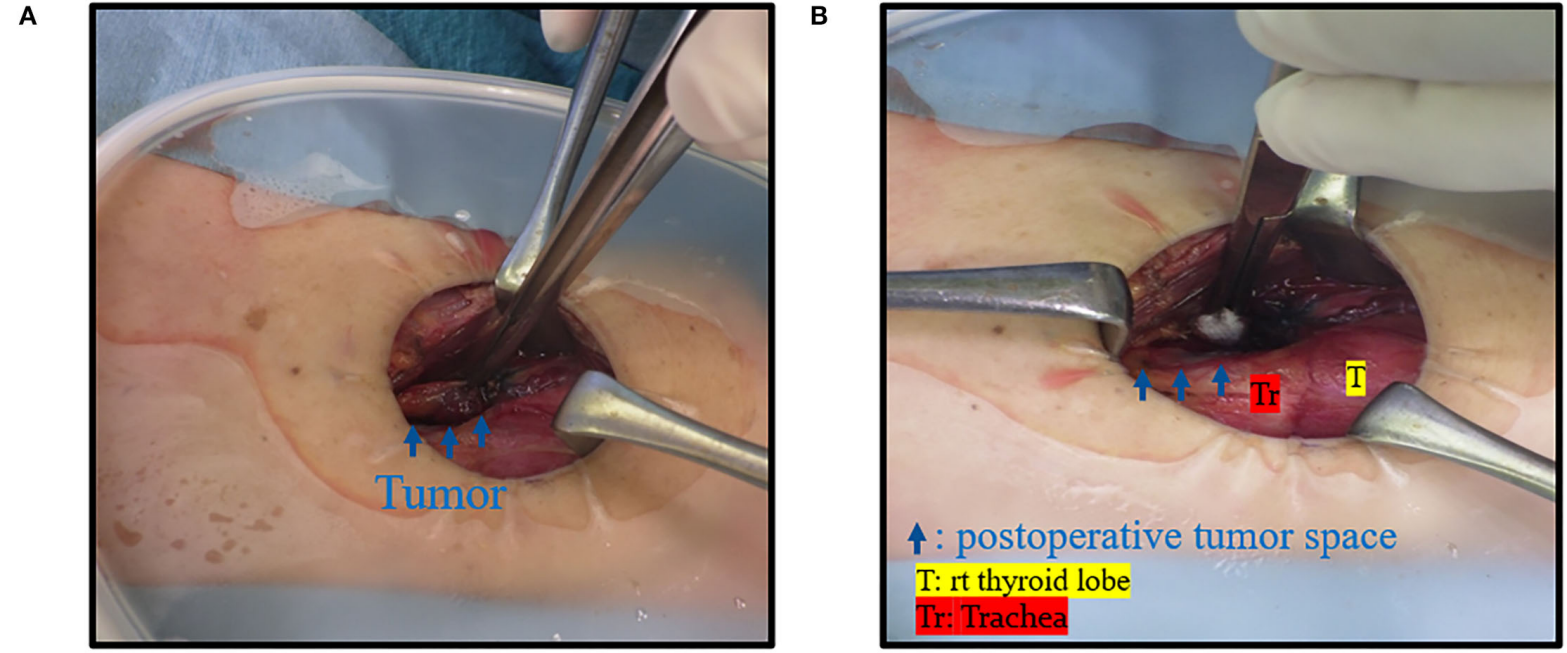
Case 8. **(A)** Intraoperative finding; there was a mass near the right trachea (blue arrow). **(B)** There was a space after the tumor was resected.

**Figure 8:**
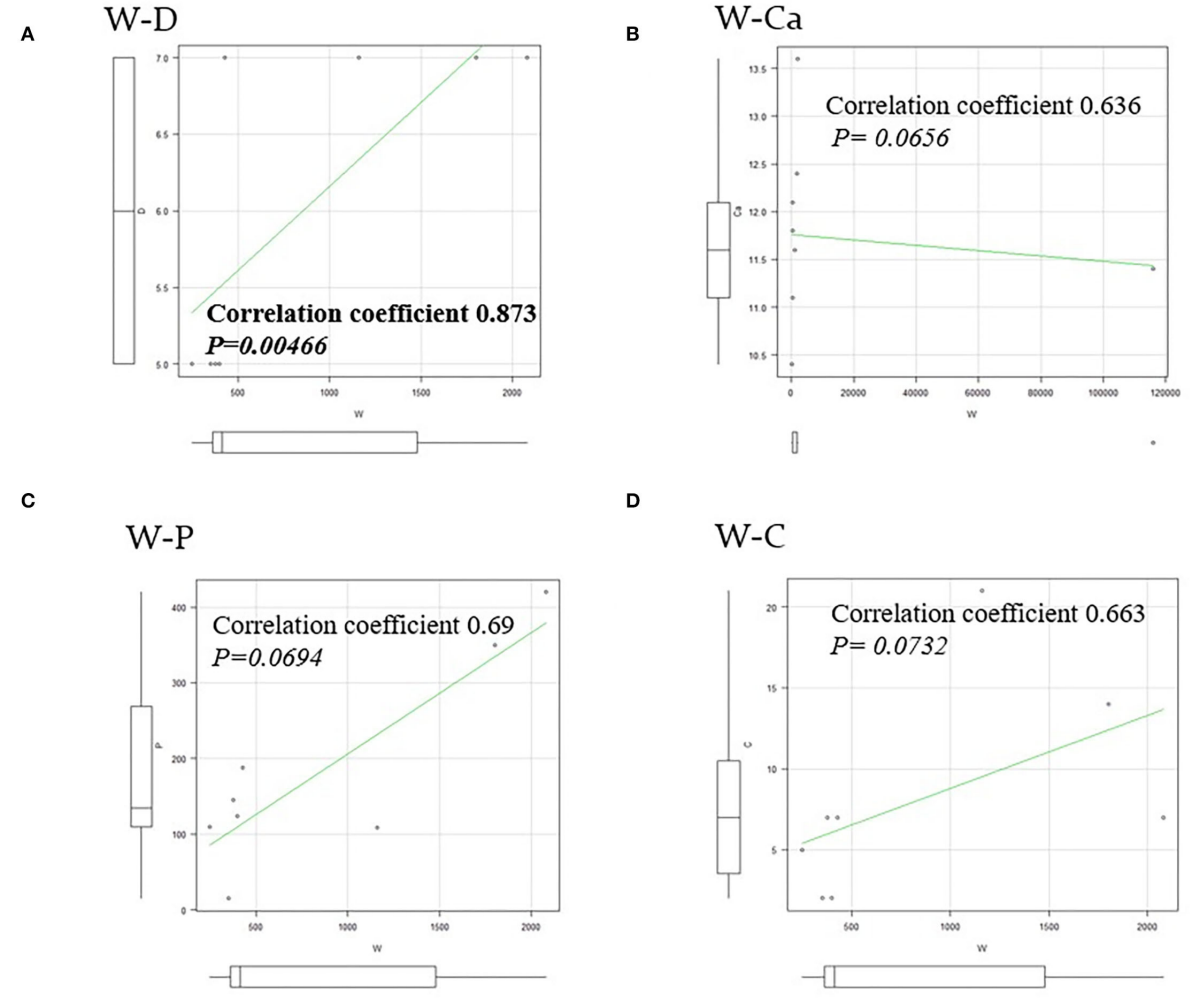
A rank correlation coefficient and differences. **(A)** There was a strong correlation between the weight of specimens and the length of hospital stay (W–D), with a rank correlation coefficient of 0.873, and differences at a *p* = 0.00466 were considered significant. **(B–D)** There was no correlation between the weight of specimens and the preoperative corrected Ca (W–Ca), the preoperative i-PTH (W–P), and the postoperative hypocalcemia course for normalized period (W–C).

There were three males and seven females (age range, 44–80 years; average age, 66.1 years). Seven patients presented with osteoporosis and/or psychiatric symptoms with biochemical hypercalcemia. Two patients presented with ureter stone-type hypercalcemia and one patient presented with bone pain, a brown tumor, and upper extremity weakness with bone-type hypercalcemia. Table 1 summarizes the results of laboratory tests showing hypercalcemia and hyperparathyroidism. A cervical neck ultrasound was performed in all patients and showed a focus in 8 patients. Cervicothoracic enhanced computed tomography (CT) was performed in all patients and showed a focus in nine patients. ^99m^Tc-MIBI scintigraphy was performed in eight patients and showed uptake in six patients. On topographic evaluation, ^99m^Tc-MIBI (late phase) showed right thyroid and anterior mediastinal washout staining and a cervicothoracic enhanced CT scan showed a focus between the right brachiocephalic artery and superior *vena cava* (Figures 3A,B). Cervicothoracic and multi-planer reconstruction (MPR) enhanced CT scan showed a focus in the left lower thyroid, and a cervical neck ultrasound showed a left lower thyroid mass (Figures 4A–C). Cervicothoracic and MPR enhanced CT scan showed a focus near the cervical esophagus, and a cervical neck ultrasound showed a mass near the cervical esophagus (Figures 5A,B). Cervicothoracic and MPR enhanced CT scan showed a focus near the right trachea and a cervical neck ultrasound showed a focus near the right trachea (Figures 6A–C). The choice of surgical approach was made based on the difficulties of dissection, which were predictable based on morphologic assessment and location of the lesion in the mediastinum. We performed a neck dissection and thoracotomy in one patient, a thoracoscopy in one patient, a focused surgical approach in seven patients (including four patients with indigo carmine injection; Figures 5–7), and surgery with a bilateral approach in one patient. The lesion was localized in the anterior mediastinum (12), on the dorsal aspect of the thoracic esophagus (13), between the right brachiocephalic artery and superior *vena cava*, in the left thyroid lobe, near the cervical esophagus, between the heads of the right sternocleidomastoid muscle, and in the left pyriform fossa in one patient, and near the right trachea in two patients. One patient had double parathyroid adenomas that were located in the left lower parathyroid gland and subcutaneous sternoclavicular joint. To minimize the risk of failure and reoperation, we performed a PTH assay 1 h after removal of the specimen. The intraoperative monitoring was considered satisfactory if there was a decrease in the i-PTH concentration of at least 50% compared to baseline. We noted a variable decrease in the i-PTH concentration between 45 and 80% compared to baseline levels. **Table 3** summarizes the advantages and disadvantages of the intraoperative PTH assay and indigo carmine injection for EPA surgery compared to conventional surgery. The pathologic examination of all resected parathyroid tumors measured between 350 and 116,000 mg (Table 1). The pathohistological diagnosis was a parathyroid adenoma in all 10 cases. The postoperative course was uneventful except for a mild postoperative hypocalcemia in all cases. The management of hypocalcemia required medication with oral calcium and active vitamin D. The hospital stay was between 5 and 10 days. There was a strong correlation between the weight of specimens and the length of hospital stay, with a rank correlation coefficient of 0.873, and differences at a *p* = 0.00466 were considered significant [excluding the variant tumor volume (116,000 mg)] (Figure 8). There was no correlation between the weight of the specimens and the preoperative corrected calcium level, i-PTH, phosphorous, ALP, and creatinine levels, and the postoperative hypocalcemia course.

**Table 1:** General characteristics of our ectopic parathyroid adenoma patients.

				**Biochemistry**					**Diagnostic imaging**									
**No**	**Age (y.o)**	**Sex**	**Clinical Presentation (mg/dl)**	**Corrected Ca (pg/ml)**	**i-PTH**	**Phosphorous (mg/dl)**	**ALP (U/l)**	**Creatinine (mg/dl)**	**Cervical ultrasound**	**3D enhanced CT#**	**Sestamibi- MIBI**	**Tumor location**	**Surgical approach weight (mg)**	Tumor course^**^	**Post operative**	**Hospital stay (days)**	**Post operative complication**	Outcome duration (years)^***^
1	59	Male	Bone pain brown tumor fatigue and arm muscle weakness	11.4	420	2.1	325	1.12	Large cervical mass	Mediastinal cystic mass	Anterior mediastinal PA thoracotomy	Anterior mediastinal	Neck and	116,000	Hypocalcemia after 2 months	10	TRLNP	Dead 5
2	44	Female	Ureter stones type	12.1	188	2.2	110	0.65	Uncertain	Posterior mediastinal PA	Posterior mediastinal PA	Dorsal side of thoracic esophagus	Video-assisted thoracoscope	428	Hypocalcemia after 1 weeks	7	None	Alive 12
3	66	Female	Biochemical type osteroprosis	12.4	350	2.3	121	0.98	Between rt brachioceohalic artery and superior vena cava	Between rt brachioceohalic artery and superior vena cava	Anterior mediastinal PA	Between rt brachioceohalic artery and superior vena cava	Focus	1,800	Hypocalcemia after 2 weeks	7	None	Alive 6
4	62	Female	Biochemical type osteroprosis	11.8	145	2.5	112	1.02	lt lower thyroid	lt thyroid in lower anterior	Intra-lt thyroid	Intra-left thyroid	Focus	377	Hypocalcemia after 1 weeks	5	None	Alive 5
5^*^	61	Female	Biochemical type osteroprosis	11.6	109	2.5	86	0.78	lt lower parathyroid and subcutaneous sternoclavicular joint	lt lower parathyroid and subcutaneous sternoclavicular joint	lt lower parathyroid and sternoclavicular joint	lt lower parathyroid and subcutaneous sternoclavicular joint	Focus	970, 1,160	Hypocalcemia after 3 weeks	7	None	Alive 4
6	80	Female	Biochemical type osteroprosis	10.4	110	3.5	95	1.15	Near cervical esophagus	near cervical esophagus	Uncertain	Near cervical esophagus	Focus dye method	250	Hypocalcemia after 5 days	5	None	Alive 3
7	80	Male	Biochemical type Psychiatric symptoms (violent behavior)	13.6	420	2.2	124	1.21	Uncertain	Uncertain	Uncertain	Between rt sternocleidomastoid muscles	Bilateral	2080	Hypocalcemia after 1 weeks	7	TRLNP	Alive 3
8	80	Female	Biochemical type osteroprosis	11.1	124	3.4	98	0.96	Near rt trachea	Near rt trachea	Not performed	Near rt trachea	Focus dye method	400	Hypocalcemia after 2 days	5	None	Alive 2
9	66	male	Recurrent ureter stones osteroprosis	11.1	150	3.8	76	0.75	lt pear-shaped fossa	lt upper parathyroid	Not performed	lt pear-shaped fossa	Focus dye method	350	Hypocalcemia after 2 days	5	None	Alive 1
10	63	Female	Biochemical type osteroprosis	11.0	181	3.5	84	0.69	rt & lt near trachea	rt & lt near trachea	rt near trachea	rt near trachea	Focus dye method	250	Hypocalcemia after 2 days	5	None	Alive 0.5

* *Double adenoma case (i-PTH: intact-parathyroid hormone, PA, parathyroid adenoma; TRLNP, transient recurrent laryngeal nerve palsy)*,

** *Normalised period*,

*** *Duration of follow up, # CT (computed tomography)*.

## Discussion

The EPAs have a variable incidence according to many studies (3, 14, 15). Phitayakorn et al. (16), in a series of 231 patients who underwent surgery for hyperparathyroidism, reported that 16% were ectopic lesions, among which 3% were intra-thymic (16). In our study, 139 patients underwent surgery for hyperparathyroidism due to parathyroid adenomas with equivalent results [9.7% (*n* = 14) ectopic lesions among which 3.0% (*n* = 4) were intra-thymic]. The caudal parathyroid glands and thymus are embryologically-derived from the third branchial pouch before migration to the cervical and mediastinal levels, respectively (17). This migration may explain the possibility of supernumerary parathyroid and ectopic glands, especially in the thoracic cavity (3). The patients with EPA are among those with PHPT diagnosed based on blood tests. In the presence of clinical signs related to phosphocalcic metabolic disorders, the clinical and radiologic examinations of the neck should search for the possibility of a cervical neck mass in relation to a parathyroid adenoma. Unfortunately, in the majority of patients these radiologic investigations are not helpful. In the current study, patients with persistent clinical and biological signs associated with hypercalcemia had cervical neck ultrasound and cervicothoracic enhanced CT scan performed, which demonstrated the lesion in the mediastinum in 8 and 9 patients, respectively. Thus, to support the diagnosis, ^99m^Tc-MIBI scintigraphy was performed in 8 patients and showed uptake in 6 patients. Indeed, sestamibi scintigraphy is currently considered the technique of choice because it has a sensitivity of approximately 95% and better image quality than a CT scan with a lower radiation exposure (3, 18, 19). According to Rousseau et al. (1), in the presence of a parathyroid adenoma, surgical resection of hyperfunctioning tissue is the gold standard. The current trend is to offer surgery to all patients in whom the diagnosis of PHPT is made, which includes asymptomatic patents regardless of age (1, 3, 20, 21). Table 2 shows the indications for surgery in the treatment of PHPT based on the 2014 guidelines for the management of asymptomatic PHPT (22). The surgery has been recommended in older patients meeting criteria for parathyroidectomy if medically stable with no contraindications for surgery and/or the benefits of surgery outweigh the risks. The advancing age is associated with greater morbidity, intra- and post-operative complications, and a higher mortality rate following parathyroidectomy (23). The guidelines recommend age below 50 years as an indication for surgery in the treatment of primary hyperthyroidism (22, 24). In contrast, several studies have shown adverse effects of hyperparathyroidism, as follows: An increased risk of premature death; hypertension; myocardial hypertrophy; mental disorders; and bone loss, especially in elderly women (1, 3, 23, 25). In our series, we operated on 139 patients for hyperparathyroidism with parathyroid adenomas. The average age of the patients was 59.6 years. There were three males and seven females (age range, 44–80 years; average age, 66.1 years) with EPAs. Compared with the patients below 50 years of age who had indications for surgery based on the guidelines, the patients who underwent surgery tended to be older. In Japan, the declining birth rate and aging population are social problems, and it is thought that the age of the surgical candidates has been increasing. Whatever the intended surgical technique, preoperative localization of the lesion is often difficult even though technical investigations are becoming more sophisticated. To minimize the risk of leaving hyperfunctioning tissue, and therefore reoperation, we initiated the use of an intraoperative PTH assay. The goal is to achieve a concentration reduction to 50% of baseline. This is an old method, but still valuable in our context because no case of recurrence was noted in our study. Hypocalcemia is an ever-present postoperative complication. According to Rousseau et al. (1), the absence of hypocalcemia raises the possibility of incomplete resection of tumoral tissue. The signs of complete resection that are most commonly found are clinical neuromuscular excitability with paresthesias, cramps, and/or tetany. At a more advanced stage, there is impaired consciousness, seizures, and laryngospasm or bronchospasm (1, 3). The preferred treatment of PHPT is surgical. The patient should only be treated if symptomatic because serum calcium levels return to normal levels on the fourth or fifth day postoperatively (1, 3). In our study, no major postoperative complications were observed in all 139 patients who underwent a parathyroidectomy. The postoperative complications were absent in four patients treated with the dye method, and transient recurrent laryngeal nerve palsy occurred in two of six patients treated without the dye method. The postoperative follow-up period was 0.5–12 years; one patient died of another disease, nine patients survived, and no patients had a postoperative recurrence. As shown in Table 3, if EPAs are stained with dye preoperatively, the EPAs can be directly detected and removed in a stress-free surgical environment without unnecessary site searches. In contrast, there was a strong correlation between the weight of specimens and the length of hospital stay, with a rank correlation coefficient of 0.873, and differences at a *p* = 0.00466 were considered significant (Figure 8). All male patients with an EPA had severe secondary osteoporosis in our series (Table 1). Caution should be exercised in the case of large parathyroid adenomas and in the elderly because osteoporosis is a complication, even in males. Bone mineral density measurement is important pre- and post-operatively in patients with hyperparathyroidism. We performed surgery with a focused approach in four patients after injecting indigo carmine (Figures 5–7). Although there was a limited number of cases, the indigo carmine injection method has the advantage of a stress-free surgical procedure because localization of the EPA can be easily confirmed intraoperatively. The convenience of the indigo carmine injection method will be confirmed by increasing the number of cases in the future. EPAs are rare and the mediastinal location in varying sites makes management challenging. A combination of cervical neck ultrasound, contrast-enhanced CT, and scintigraphy may be useful for localization of EPAs. Despite the performance of these increasingly sophisticated investigative techniques, the surgeon must perform an accurate and meticulous dissection to minimize the risk of leaving behind a hyperfunctioning parathyroid gland. The intraoperative PTH assay and indigo carmine injection intraoperatively are valuable tools that facilitate appropriate surgical management.

**Table 2:** Indication for surgery in the treatment of primary hyperthyroidism.

1) Age <50 years.
2) Serum calcium > 1mg/dL or 0.25 mmol/L of the upper limit of the reference interval for total calcium and > 0.12 mmol/L for Ca^2+^.
3) BMD T-sore < = 2.5 at the lumbar spine, femoral neck, the total hip, or the 1/3 radius for postmenopausal women or males >50yr.
A prevalent low-energy fracture (i.e., in the spine) is also considered an indication for surgery, which requires a routine X ray of the thoracic and lumbar spine (or vertebral fracture assessment by DXA).
4) A glomerular filtration rate (GFR) of <0 ml/min. Further evaluation of asymptomatic patients with renal imaging (X ray, CT or ultrasound) in order to detect silent kidney stones or nephrocalcinosis is advised. A complete urinary stone risk profile should be performed in those individuals whose urinary calcium excretion is >400 mg/day. If stone(s), nephrocalcinosis, or high stone risk is determined, surgery should be recommended.
From 2014 Guidelines for the management of asymptomatic primary hyperthyroidism: summary statement from the Fourth International Workshop.

**Table 3:** Advantages and disadvantages of intraoperative parathyroid. Hormone assay and indigo carmine injection for EPA and conventional surgery.

**Intraoperative parathyroid hormone assay**	
Advantage	Minimizes the risk of failure and reoperation.
Disadvantages	It is necessary to establish a laboratory for intraoperative measurement of PTH.
	Cooperation between the anesthesiologist and operating room staff is nesessary for frequent blood sampling.
**Indigo carmine injection**	
Advantage	A stress-free surgical procedure because localization of the EPA can be easily confirmed intraoperatively.
Disadvantage	The indigo carmine injection set and the ultrasonic testing device must be prepared before the operation, which is rather complicated.

## Data Availability Statement

The original contributions presented in the study are included in the article/supplementary material, further inquiries can be directed to the corresponding author/s.

## Ethics Statement

The studies involving human participants were reviewed and approved by International University Health and Welfare Ichikawa Hospital and Niizashiki Central General Hospital. The patients/participants provided their written informed consent to participate in this study. Written informed consent was obtained from the individual(s) for the publication of any potentially identifiable images or data included in this article.

## Author Contributions

SK, HO, NK, and MahK performed data collections and data analysis. MakK developed the manuscript and all the remaining authors provided critical edits to the final draft. All authors contributed to the study design, critically interpreted all data analyses, and read and approved the final manuscript.

## Conflict of Interest

The authors declare that the research was conducted in the absence of any commercial or financial relationships that could be construed as a potential conflict of interest.

## Publisher's Note

All claims expressed in this article are solely those of the authors and do not necessarily represent those of their affiliated organizations, or those of the publisher, the editors and the reviewers. Any product that may be evaluated in this article, or claim that may be made by its manufacturer, is not guaranteed or endorsed by the publisher.
